# Molecular Confirmation of *Anopheles stephensi* Mosquitoes in the Al Hudaydah Governorate, Yemen, 2021 and 2022

**DOI:** 10.3201/eid3007.240331

**Published:** 2024-07

**Authors:** Methaq Assada, Mohammed Al-Hadi, Mohammed A. Esmail, Jamil Al-Jurban, Abdulsamad Alkawri, Arif Shamsan, Payton Terreri, Jeanne N. Samake, Adel Aljasari, Abdullah A. Awash, Samira M. Al Eryani, Tamar E. Carter

**Affiliations:** Ministry of Health, Sana’a, Yemen (M. Assada, M. Al-Hadi, M.A. Esmail, J. Al-Jurban, A. Alkawri, A. Shamsan);; Baylor University, Waco, Texas, USA (P. Terreri, J.N. Samake, T.E. Carter);; World Health Organization Country Office, Sana’a (A. Aljasari, A.A. Awash);; World Health Organization, Cairo, Egypt (S.M. Al Eryani)

**Keywords:** *Anopheles stephensi*, genotyping, invasive species, malaria, vector-borne infections, parasites, mosquitoes, Yemen

## Abstract

We detected malaria vector *Anopheles stephensi* mosquitoes in the Al Hudaydah governorate in Yemen by using DNA sequencing. We report 2 cytochrome *c* oxidase subunit I haplotypes, 1 previously found in Ethiopia, Somalia, Djibouti, and Yemen. These findings provide insight into invasive *An. stephensi* mosquitoes in Yemen and their connection to East Africa.

Malaria remains a major threat to global health with ≈247 million cases reported in 2021 (*1*). An invasive malaria vector, *Anopheles stephensi* mosquito, has emerged in Africa; the first detection was in Djibouti in 2012 and was followed by detections in Ethiopia, Somalia, Sudan, Nigeria, Ghana, Kenya, and Eritrea (*2*) (Appendix, https://wwwnc.cdc.gov/EID/article/30/7/24-0331-App1.pdf). With growing evidence of *An. stephensi* mosquito resistance to multiple classes of insecticides (*3*,*4*), its association with a recent malaria outbreak (*5*), and genomic evidence that outbreak sites may also be central locations for *An. stephensi* mosquito travel to new areas (*6*), concerns are growing about the status and spread of this mosquito species in the Mediterranean region.

In the Arabian Peninsula, the geographic distribution of *An. stephensi* mosquitoes is unclear. Previous field investigations and predictive modeling indicate native populations exist in the northeastern coastal region along the Persian Gulf and inland in countries including Saudi Arabia (*7*,*8*). The status of *An. stephensi* mosquitoes is important in Yemen, where an increase in malaria cases was reported in the city of Aden beginning in 2017, although a link to *An. stephensi* mosquitoes has not been investigated (*9*). The first report of *An. stephensi* mosquitoes in Yemen occurred in Aden in 2021 and was confirmed with molecular analysis in 2023 (*2*,*10*). Recent retrieval of an unpublished entomological survey report indicated *An. stephensi* mosquitoes were present within the Al Zuhra district in the Al-Hudaydah governorate in 2000 (World Health Organization Yemen, unpub. data). No documentation of *An. stephensi* mosquitoes before 2000 or in later entomological surveys was found until the recent detection in 2021. Little is known about the distribution and characteristics of this vector in western governorates where the highest prevalence of malaria in Yemen is documented (*9*). *An. stephensi* mosquitoes were reported for the first time in the Ad Dahi district in December 2021 and in the Zabid district in March 2022, both within the Al Hudaydah governorate (*2*). *An. stephensi* mosquitoes have recently been found in multiple suburban areas in the Tehama coastal plain region (*11*). In this study, we characterize the genetic diversity of *An. stephensi* mosquitoes found during vector surveillance in the Al Hudaydah governorate.

## The Study

**Figure 1:**
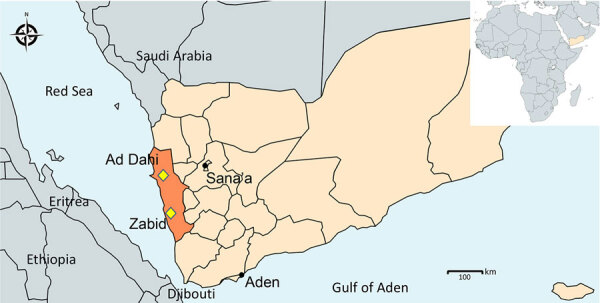
Locations in Yemen where invasive *Anopheles stephensi* mosquitoes were detected in 2021 and 2022 (yellow diamonds). Map was created by using MapChart (https://www.mapchart.net).

We analyzed immature mosquitoes collected from 2 semiurban locations, Ad Dahi and Zabid districts (Figure 1; Appendix). We collected the Ad Dahi district specimens over the course of a single day in December 2021 while conducting *Aedes* surveillance during a dengue fever outbreak. The Zabid district specimens were collected during monthlong *Anopheles* surveillance in March 2022. Potential breeding containers surveyed included open concrete ponds and cement water tanks near block factories, washing basins in mosques, and car washing sites. Mosquitos used in this study were collected as immature specimens by using the dipping method. We reared them to adults in field insectaries and then identified them by using updated morphological keys (*12*). The specimens we morphologically identified as *An. stephensi* mosquitoes were preserved with silica gel. We sent a subsample of specimens to Baylor University (Waco, Texas, USA) for molecular analysis.

For species identification, we analyzed 2 loci, cytochrome *c* oxidase subunit 1 (COI) and internal transcribed spacer 2 (ITS2), by using previously described protocols (*13*). We used an *An.* *stephensi*–specific endpoint PCR as previously described (*14*). We conducted ITS2- and COI-targeted DNA sequencing, then used BLAST (https://blast.ncbi.nlm.nih.gov) and conducted phylogenetic analysis to determine species identification and evaluate the level of genetic diversity (Appendix).

Most of the *Anopheles* larvae were collected from cement water tanks found outside of homes, which were like other invasive settings found in eastern Ethiopia and Aden (*4*,*10*). We identified 41 mosquitoes morphologically as *An. stephensi*, 7 from Ad Dahi and 34 from Zabid City. Of the 41 specimens, all 7 Ad Dahi and 32 of the Zabid City specimens were confirmed to be *An. stephensi* mosquitoes by ITS2 endpoint assay and by COI and ITS2 targeted DNA sequence analysis (Appendix). We observed no discrepancy between the endpoint assay and sequencing assay for species identification. We did not report any discrepancies with the ITS2 endpoint assay results when compared with the DNA sequence results, but incorrect identification with the endpoint assay for other populations with unreported ITS2 variation is possible. Sequencing should accompany initial implementation of an endpoint assay in newly surveyed populations to evaluate both false negatives and false positives.

**Figure 2:**
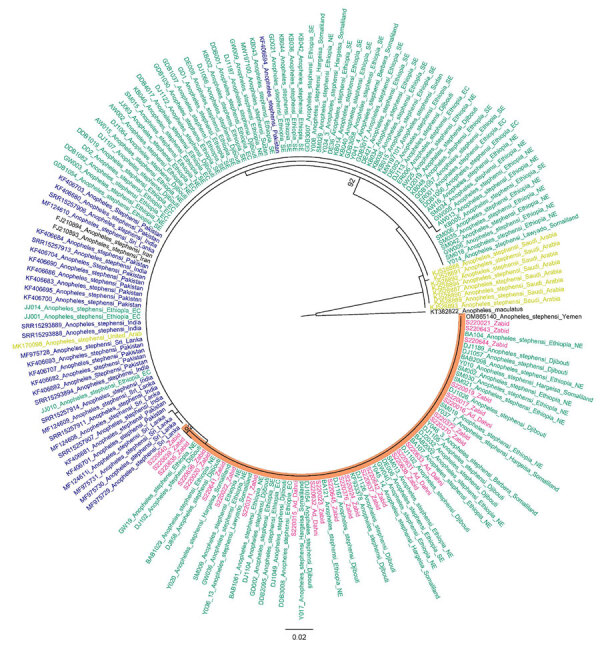
Maximum-likelihood phylogenetic analysis of cytochrome *c* oxidase subunit 1 gene sequences for *Anopheles stephensi* mosquitoes collected in Yemen. Pink indicates sequences from Yemen, blue indicates sequences from South Asia, green indicates sequences from the Horn of Africa, and yellow-green indicates sequences from the Arabian Peninsula. Orange shading indicates branch containing Yemen and Horn of Africa specimens only. Numbers along branches indicate bootstrap values. Only values >70 are shown. Scale bar indicates the number of nucleotide substitutions per site.

**Figure 3:**
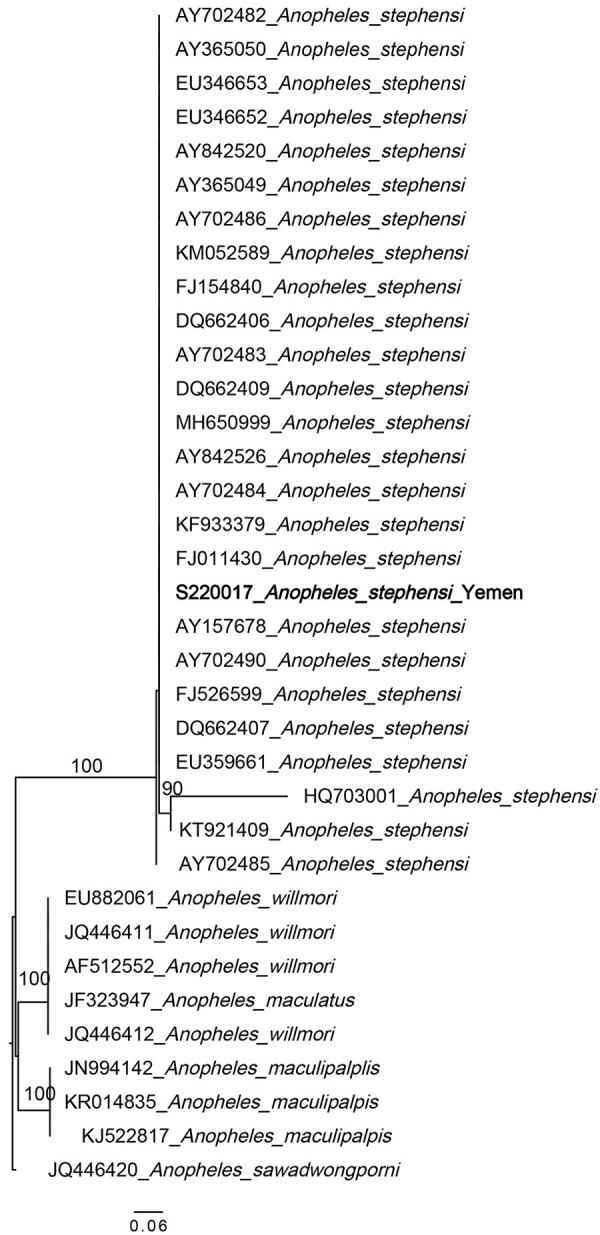
Maximum-likelihood phylogenetic analysis of *An.* internal transcribed spacer 2 DNA sequences for *Anopheles stephensi* mosquitoes collected in Yemen. Only 1 sequence (bold) is included as a representative of the single haplotype observed in the Yemen *An. stephensi* specimens. GenBank accession numbers are provided. Numbers along branches indicate bootstrap values. Only values >70 are shown. Scale bar indicates the number of nucleotide substitutions per site.

Two non–*An. stephensi* mosquitoes were recovered; 1 was identified as *Anopheles culicifacies* and 1 as *Aedes aegypti* on the basis of ITS2 and COI sequence BLAST analysis. This change from the initial morphological identification could be the result of sorting the *Anopheles* and *Aedes* mosquitoes from the same habitat or from the selection of specimens for molecular analysis in the laboratory. Phylogenetic analysis confirmed the species identification of the *An. stephensi* mosquito specimens (bootstrap = 100 for COI, bootstrap = 100 for ITS2) (Figures 2, 3).

ITS2 sequences were all identical for the *An. stephensi* mosquito specimens and BLAST analysis revealed a 100% sequence identification in GenBank. Sequence analysis of COI revealed 2 haplotypes. One haplotype (n=35) was previously reported throughout the Horn of Africa (HoA) (COIHap 3) with the highest frequency in northeastern Ethiopia, Somaliland, and Djibouti (*15*). This haplotype was also noted in a recent report on the detection of *An. stephensi* mosquitoes in Aden, Yemen (*10*). The second haplotype (n=4), differing by a single nucleotide from COIHap3, has not previously been reported (designated COIHapYem1).

These findings demonstrate the successful use of ongoing vector surveillance activities for the initial detection of *An. stephensi* mosquitoes. The detection of a common HoA COI haplotype raises questions about the relationship between the invasive *An. stephensi* mosquitoes in northern HoA and Yemen. It is possible they share a common origin or that movement of *An. stephensi* mosquitoes has occurred between the 2 regions. Sequencing of additional loci could assist with delineating their relationship. Our findings provide information about the relative timing of the Yemen introduction of *An. stephensi* mosquitoes relative to the HoA detection on the basis of several observations. Fewer haplotypes were observed in the Al Hudaydah region in comparison to northeastern Ethiopia, Somalia, and Djibouti, which may indicate a recent introduction into the Al Hudaydah region. COIHap3 has a limited geographic range in comparison to the HoA-wide Hap2, which suggests the haplotype from our study could be associated with a later introduction in HoA and potentially Yemen. As for the HoA specimens and the study in Aden, no Saudi Arabian haplotypes were detected. Unlike the HoA specimens, no South Asia haplotypes were detected. Further genomic analysis and extensive *An. stephensi* mosquito sampling in Yemen, Saudi Arabia, and other parts of the Arabian Peninsula are needed to evaluate the hypothesis of a recent introduction in Yemen relative to the HoA.

## Conclusions

Our findings provide insight into the genetic diversity of *An. stephensi* mosquito populations in Al Hudaydah governorate, Yemen. The findings also provide support for the need of long-term entomological and epidemiologic surveillance of *An. stephensi* mosquitoes on malaria transmission in the region. With the detection of *An. stephensi* mosquitoes in Yemen, concerns remain related to the status of these mosquitoes in other areas of Yemen and their relationship with the HoA *An. stephensi* mosquitoes. Additional genomic analysis should be conducted to further examine the relative timing of the introduction of *An. stephensi* mosquitoes to Yemen.

Appendix Additional information about molecular confirmation of *Anopheles stephensi* mosquitoes in the Al Hudaydah governorate, Yemen, 2021 and 2022.
