# Supplementary material for: Molecular Confirmation of Anopheles stephensi Mosquitoes in the Al Hudaydah Governorate, Yemen, 2021 and 2022
Source: Emerg Infect Dis. 2024 Jul;30(7):1467–71. doi: 10.3201/eid3007.240331 (PMC11210655; doi:10.3201/eid3007.240331)
Supplement: Appendix — Additional information about molecular confirmation of Anopheles stephensi mosquitoes in the Al Hudaydah governorate, Yemen, 2021 and 2022. [file 24-0331-Techapp-s1.pdf]

Article DOI: https://doi.org/10.3201/eid3007.240331

EID cannot ensure accessibility for supplementary materials supplied by authors. Readers who have difficulty accessing supplementary content should contact the authors for assistance.

Molecular confirmation of *Anopheles stephensi* Mosquitoes in the Al Hudaydah Governorate, Yemen, 2021 and 2022

Appendix

The invasive malaria vector, *Anopheles stephensi* mosquito, in Africa was first detected in Djibouti in 2012, and was followed by Ethiopia, Somalia, Sudan, Nigeria, Ghana, Kenya, and Eritrea over the past decade (*1*–*7*, Afrane YA, unpub. data, https://pubmed.ncbi.nlm.nih.gov/38076990).

Site descriptions

Ad Dahi, located north of Hodeida city (15° 12′ 55” N / 43° 4′ 13” E). The city has a population size of ≈21,587 and the climate is tropical with high temperatures in the summer and moderate temperatures in the winter. Zabid, one of the southernmost districts of Al Hudaydah governorate, is located in the Tehama coastal plain near the Red Sea (14°12′02.6”N, 43°19′06.6”E) in a tropical climate and a population size of 34,686.

Molecular analysis

Two loci were selected for PCR-based species identifications: cytochrome oxidase subunit 1 (COI) and internal transcribed spacer 2 (ITS2). A *stephensi*-specific PCR endpoint assay was used to identify *An. stephensi* based on amplification (presence/absence) of a portion of the ITS2 locus. The primer sequences for PCR in the ITS2 endpoint assay were 5.8SB (5′-ATCACTCGGCTCGTGGATCG-3′) and 28SC (5′- GTCTCGCGACTGCAACTG-3′) (*7*). In addition, two PCR protocols for the *ITS2* locus and the *COI* gene were implemented to generate products for sequencing. PCR was conducted as detailed in Carter et al. (*3*). The primer sequences for the ITS2 PCR for sequencing were 5.8SB (5′-ATCACTCGGCTCGTGGATCG-3′) and 28SB (5′-ATGCTTAAATTTAGGGGGTAGTC-3′) (*7*). The primer sequences for *COI* PCR were LCO1490F (5′-GGTCAACAAATCATAAAGATATTGG-3′) and HCO2198R (5′-

TAAACTTCAGGGTGACCAAAAAATCA-3′) (*8*). COI and ITS2 PCR products were sequenced using Sanger sequencing technology. Sequences were then trimmed using CodonCode Aligner (CodonCode Corporation, Centerville, MA). To avoid sequence ambiguity caused by the microsatellite loci located within ITS2 (*3*, *9*), we only considered the sequence upstream of a previous identified microsatellite. For the COI, a 317 bp region was used for phylogenetic analysis carried phytogeographically informative single nucleotide polymorphisms (SNPS) (*10*) (Carter et al. 2021). Trimmed sequences were submitted to the National Center for Biotechnology Information's (NCBI) Basic Local Alignment Search Tool (BLAST) to confirm successful amplification. The sequences were further aligned in CodonCode with previously published sequences retrieved from Genbank, and phylogenetic analyses were conducted using maximum-likelihood method with RAxML (*11*). The final trees were annotated using Figtree (Rambaut, http://tree.bio.ed.ac.uk/software/figtree/URL).

Availability of data and materials

The sequences generated in this study are available through NCBI Genbank Accession numbers PP410027, PP387837- PP387838.

Abbreviations

HoA: Horn of Africa; WHO: World Health Organization; PCR: polymerase chain reaction; ITS2: internal transcribed spacer 2; COI: cytochrome c oxidase I; NCBI: National Center for Biotechnology Information, BLAST: basic local alignment search tool; SNPs: single nucleotide polymorphisms

References

1. Sinka ME, Bangs MJ, Manguin S, Chareonviriyaphap T, Patil AP, Temperley WH, et al. The dominant *Anopheles* vectors of human malaria in the Asia-Pacific region: occurrence data, distribution maps and bionomic précis. Parasit Vectors. 2011;4:89. PubMed https://doi.org/10.1186/1756-3305-4-89

2. Faulde MK, Rueda LM, Khaireh BA. First record of the Asian malaria vector *Anopheles stephensi* and its possible role in the resurgence of malaria in Djibouti, Horn of Africa. Acta Trop. 2014;139:39–43. PubMed https://doi.org/10.1016/j.actatropica.2014.06.016

3. Carter TE, Yared S, Gebresilassie A, Bonnell V, Damodaran L, Lopez K, et al. First detection of *Anopheles stephensi* Liston, 1901 (Diptera: culicidae) in Ethiopia using molecular and morphological approaches. Acta Trop. 2018;188:180–6. PubMed https://doi.org/10.1016/j.actatropica.2018.09.001

4. Ali S, Samake JN, Spear J, Carter TE. Morphological identification and genetic characterization of *Anopheles stephens*i in Somaliland. Parasit Vectors. 2022;15:247. PubMed https://doi.org/10.1186/s13071-022-05339-y

5. Ahmed A, Pignatelli P, Elaagip A, Abdel Hamid MM, Alrahman OF, Weetman D. Invasive malaria vector *Anopheles stephensi* mosquitoes in Sudan, 2016-2018. Emerg Infect Dis. 2021;27:2952–4. PubMed https://doi.org/10.3201/eid2711.210040

6. Ochomo EO, Milanoi S, Abong'o B, Onyango B, Muchoki M, Omoke D, et al. Detection of *Anopheles stephensi* mosquitoes by molecular surveillance, Kenya. Emerg Infect Dis. 2023;29:2498–508. PubMed https://doi.org/10.3201/eid2912.230637

7. Djadid ND, Gholizadeh S, Aghajari M, Zehi AH, Raeisi A, Zakeri S. Genetic analysis of rDNA-ITS2 and RAPD loci in field populations of the malaria vector, *Anopheles stephensi* (Diptera: culicidae): implications for the control program in Iran. Acta Trop. 2006;97:65–74. PubMed https://doi.org/10.1016/j.actatropica.2005.08.003

8. Folmer O, Black M, Hoeh W, Lutz R, Vrijenhoek R. DNA primers for amplification of mitochondrial cytochrome c oxidase subunit I from diverse metazoan invertebrates. Mol Mar Biol Biotechnol. 1994;3:294–9. PubMed

9. Mishra S, Sharma G, Das MK, Pande V, Singh OP. Intragenomic sequence variations in the second internal transcribed spacer (ITS2) ribosomal DNA of the malaria vector *Anopheles stephensi.* PLoS One. 2021;16:e0253173. PubMed https://doi.org/10.1371/journal.pone.0253173

10. Carter TE, Yared S, Getachew D, Spear J, Choi SH, Samake JN, et al. Genetic diversity of *Anopheles stephensi* in Ethiopia provides insight into patterns of spread. Parasit Vectors. 2021;14:602. PubMed https://doi.org/10.1186/s13071-021-05097-3

11. Stamatakis A. RAxML version 8: a tool for phylogenetic analysis and post-analysis of large phylogenies. Bioinformatics. 2014;30:1312–3. PubMed https://doi.org/10.1093/bioinformatics/btu033
